# *N*-[2-(3,4-Dimeth­oxy­phen­yl)eth­yl]-*N*-methyl­benzene­sulfonamide

**DOI:** 10.1107/S1600536812005272

**Published:** 2012-02-29

**Authors:** Jasmine P. Vennila, D. John Thiruvadigal, E. Theboral Sugi Kamala, Helen P. Kavitha, V. Manivannan

**Affiliations:** a Department of Physics, Panimalar Institute of Technology, Chennai 602 103, India; b Department of Physics, SRM University, Kattankulathur Campus, Chennai 603 203, India; c Department of Physics, Easwari Engineering College, Ramapuram, Chennai 600 089, India; d Department of Chemistry, SRM University, Ramapuram Campus, Chennai 600 089, India; e Department of Research and Development, PRIST University, Vallam, Thanjavur 613 403, India

## Abstract

In the title compound, C_17_H_21_NO_4_S, the phenyl and dimeth­oxy­phenyl rings are almost perpendicular to each other, making a dihedral angle of 82.57 (5)°. The structure is stabilized by inter­molecular C—H⋯O inter­actions and the packing is further enhanced by C—H ⋯π inter­actions.

## Related literature

For the biological activity of sulfonamide derivatives, see: Zareef *et al.* (2007 ▶); Pomarnacka & Kozlarska-Kedra (2003 ▶); Siddiqui *et al.* (2007 ▶); Gennarte *et al.* (1994 ▶). For standard bond distances, see: Allen *et al.* (1987 ▶). For geometric parameters, see: Khan *et al.* (2010 ▶). For asymmetry parameters, see: Nardelli (1983 ▶).
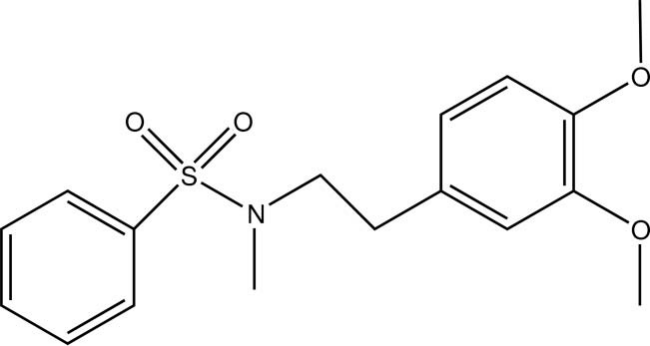


## Experimental

### 

#### Crystal data

C_17_H_21_NO_4_S
*M* *_r_* = 335.41
Monoclinic, 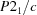

*a* = 9.9383 (3) Å
*b* = 14.6494 (4) Å
*c* = 12.0097 (3) Å
β = 108.535 (1)°
*V* = 1657.80 (8) Å^3^
*Z* = 4
Mo *K*α radiation
μ = 0.22 mm^−1^
*T* = 293 K
0.40 × 0.40 × 0.30 mm

#### Data collection

Bruker Kappa APEXII CCD diffractometer
Absorption correction: multi-scan (*SADABS*; Bruker, 2004 ▶) *T*_min_ = 0.879, *T*_max_ = 0.938
20493 measured reflections
4353 independent reflections
3342 reflections with *I* > 2σ(*I*)
*R*_int_ = 0.028

#### Refinement

*R*[*F* ^2^ > 2σ(*F* ^2^)] = 0.039
*wR*(*F* ^2^) = 0.115
*S* = 1.00
4353 reflections
212 parameters
H-atom parameters constrained
Δρ_max_ = 0.27 e Å^−3^
Δρ_min_ = −0.30 e Å^−3^

### 

Data collection: *APEX2* (Bruker, 2004 ▶); cell refinement: *APEX2* and *SAINT* (Bruker, 2004 ▶); data reduction: *SAINT* and *XPREP* (Bruker, 2004 ▶); program(s) used to solve structure: *SHELXS97* (Sheldrick, 2008 ▶); program(s) used to refine structure: *SHELXL97* (Sheldrick, 2008 ▶); molecular graphics: *ORTEP-3* (Farrugia, 1997 ▶) and *Mercury* (Macrae *et al.*, 2008 ▶); software used to prepare material for publication: *PLATON* (Spek, 2009 ▶).

## Supplementary Material

Crystal structure: contains datablock(s) I, global. DOI: 10.1107/S1600536812005272/sj5187sup1.cif

Structure factors: contains datablock(s) I. DOI: 10.1107/S1600536812005272/sj5187Isup2.hkl

Supplementary material file. DOI: 10.1107/S1600536812005272/sj5187Isup3.cml

Additional supplementary materials:  crystallographic information; 3D view; checkCIF report

## Figures and Tables

**Table 1:** Hydrogen-bond geometry (Å, °) *Cg*2 is the centroid of the phenyl plane C10–C15.

*D*—H⋯*A*	*D*—H	H⋯*A*	*D*⋯*A*	*D*—H⋯*A*
C14—H14⋯O2^i^	0.93	2.60	3.380 (2)	142
C8—H8*A*⋯O3^ii^	0.97	2.71	3.620 (2)	156
C4—H4⋯O1^iii^	0.93	2.65	3.369 (2)	135
C3—H3⋯*Cg*2^iv^	0.93	2.91	3.661 (2)	139
C6—H6⋯*Cg*2^ii^	0.93	3.05	3.827 (8)	123

Symmetry codes: (i) 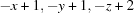; (ii) 
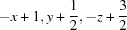
; (iii) 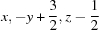; (iv) 
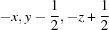
.

**Fig. 1.**
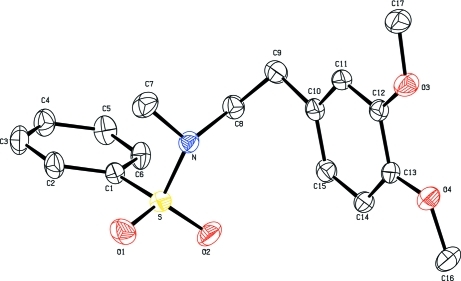
The molecular structure of (I) with 30% probability displacement ellipsoids.

**Fig. 2.**
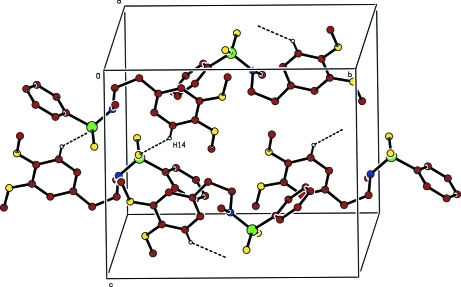
The packing of the molecules viewed along b axis.

## supplementary crystallographic information

### Comment

Sulfonamides exhibit a wide vareity of pharmocological activities such as antibacterial, antitumour, anti-carbonic anhydrase, diuretic hypoglycaemic, antithyroid and protease inhibitory activity. Sulfonamides have also been used clinically as antimalarial agents (Zareef *et al.*, 2007). Sulfonamide derivatives are known to exhibit anticancer and HIV activities (Pomarnacka & Kozlarska-Kedra, 2003). They are also used as anti-convulsants (Siddiqui *et al.*, 2007) and for the treatment of inflammatory rheumatic & non-rheumatic processes (Gennarte *et al.*, 1994).

Fig. 1 shows the structure of compound (I). Bond lengths are comparable with other reported values (Allen *et al.*, 1987).

In the title compound (I) the geometric parameters are similar with those of a similar structure (Khan *et al.*, 2010). The phenyl rings are almost perpendicular to each other making a dihedral angle of 82.57 (5)°. The sum of bond angles around N atom [345.31°] indicates *sp*^2^ hybridization. The asymmetry parameters for the phenyl rings C1—C6 and C10—C15 are given by Δ_s_ (C2) = 0.46°, Δ_2_ (C1) = 0.28°, Δ_s_ (C12) = 0.32° and Δ_2_ (C11) = 0.22° [Nardelli, 1983]. The crystal packing shows that the molecules are linked into a three dimensional framework through intermolecular C14—H14···02, C8—H8A···03 and C4—H4···01 hydrogen bonds . The packing is further stabilized by C3—H3···*Cg*(2) and C6—H6···*Cg*(2) C—H··· π interactions. [*Cg*(2) is the centroid of the C10—C15 ring], with a distances 3.661 (2)Å and 3.827 (8) Å respectively.

### Experimental

2-(3,4-dimethoxyphenyl)-*N*-methyl ethanamine (5 mmol) was dissolved in dichloromethane (20 ml) in a round bottom flask. To this, triethylamine (10.2 mmol) was added with stirring for 5 minutes. Then benzenesulfonyl chloride (51 mmol) was added into the reaction mixture and heated to 50 °C for 6 hrs. After cooling the reaction mixture to the ambient temperature, it was added to water (20 ml). The aqueous layer was separated. The ethyl acetate layer was washed twice with 10% sodium chloride solution. The organic layer was dried over 2 g of anhydrous sodium sulfate and filtered. The filtrate was evaporated under vacuum to isolate the crude compound. Recrystallization of the compound using ethyl acetate and hexane mixture yielded diffraction quality crystals.

### Refinement

H atoms were placed in idealized positions and allowed to ride on their parent atoms, with C–H = 0.93 or 0.96Å and *U*_iso_(H)= 1.2–1.5*U*_eq_(C).

### Crystal data

**Table:**

C_17_H_21_NO_4_S	*F*(000) = 712
*M**_r_* = 335.41	*D*_x_ = 1.344 Mg m^−^^3^
Monoclinic, *P*2_1_/*c*	Mo *K*α radiation, λ = 0.71073 Å
Hall symbol: -P 2ybc	Cell parameters from 7936 reflections
*a* = 9.9383 (3) Å	θ = 2.2–29.0°
*b* = 14.6494 (4) Å	µ = 0.22 mm^−^^1^
*c* = 12.0097 (3) Å	*T* = 293 K
β = 108.535 (1)°	Block, colourless
*V* = 1657.80 (8) Å^3^	0.40 × 0.40 × 0.30 mm
*Z* = 4	

### Data collection

**Table:**

Bruker Kappa APEXII CCD diffractometer	4353 independent reflections
Radiation source: fine-focus sealed tube	3342 reflections with *I* > 2σ(*I*)
Graphite monochromator	*R*_int_ = 0.028
ω and φ scans	θ_max_ = 29.2°, θ_min_ = 2.2°
Absorption correction: multi-scan (*SADABS*; Bruker, 2004)	*h* = −13→8
*T*_min_ = 0.879, *T*_max_ = 0.938	*k* = −19→19
20493 measured reflections	*l* = −16→14

### Refinement

**Table:**

Refinement on *F*^2^	Secondary atom site location: difference Fourier map
Least-squares matrix: full	Hydrogen site location: inferred from neighbouring sites
*R*[*F*^2^ > 2σ(*F*^2^)] = 0.039	H-atom parameters constrained
*wR*(*F*^2^) = 0.115	*w* = 1/[σ^2^(*F*_o_^2^) + (0.0586*P*)^2^ + 0.383*P*] where *P* = (*F*_o_^2^ + 2*F*_c_^2^)/3
*S* = 1.00	(Δ/σ)_max_ = 0.001
4353 reflections	Δρ_max_ = 0.27 e Å^−^^3^
212 parameters	Δρ_min_ = −0.30 e Å^−^^3^
0 restraints	Extinction correction: *SHELXL97* (Sheldrick, 2008), Fc^*^=kFc[1+0.001xFc^2^λ^3^/sin(2θ)]^-1/4^
Primary atom site location: structure-invariant direct methods	Extinction coefficient: 0.0028 (10)

### Special details

**Table:**

Geometry. All e.s.d.'s (except the e.s.d. in the dihedral angle between two l.s. planes) are estimated using the full covariance matrix. The cell e.s.d.'s are taken into account individually in the estimation of e.s.d.'s in distances, angles and torsion angles; correlations between e.s.d.'s in cell parameters are only used when they are defined by crystal symmetry. An approximate (isotropic) treatment of cell e.s.d.'s is used for estimating e.s.d.'s involving l.s. planes.
Refinement. Refinement of *F*^2^ against ALL reflections. The weighted *R*-factor *wR* and goodness of fit *S* are based on *F*^2^, conventional *R*-factors *R* are based on *F*, with *F* set to zero for negative *F*^2^. The threshold expression of *F*^2^ > σ(*F*^2^) is used only for calculating *R*-factors(gt) *etc*. and is not relevant to the choice of reflections for refinement. *R*-factors based on *F*^2^ are statistically about twice as large as those based on *F*, and *R*- factors based on ALL data will be even larger.

### Fractional atomic coordinates and isotropic or equivalent isotropic displacement parameters (Å^2^)

**Table:**

	*x*	*y*	*z*	*U*_iso_*/*U*_eq_	
C1	0.11829 (14)	0.66352 (10)	0.73921 (12)	0.0389 (3)	
C2	−0.02018 (16)	0.69298 (11)	0.71549 (15)	0.0503 (4)	
H2	−0.0811	0.6630	0.7481	0.060*	
C3	−0.06711 (18)	0.76743 (13)	0.64283 (16)	0.0590 (4)	
H3	−0.1597	0.7881	0.6275	0.071*	
C4	0.02163 (19)	0.81095 (11)	0.59322 (15)	0.0557 (4)	
H4	−0.0108	0.8607	0.5440	0.067*	
C5	0.15918 (18)	0.78080 (11)	0.61644 (16)	0.0548 (4)	
H5	0.2191	0.8102	0.5822	0.066*	
C6	0.20867 (15)	0.70752 (10)	0.68983 (15)	0.0477 (4)	
H6	0.3019	0.6878	0.7060	0.057*	
C7	0.01095 (18)	0.45745 (13)	0.66936 (16)	0.0588 (4)	
H7A	−0.0252	0.4999	0.6060	0.088*	
H7B	−0.0454	0.4605	0.7210	0.088*	
H7C	0.0068	0.3968	0.6383	0.088*	
C8	0.25582 (17)	0.47960 (10)	0.66388 (14)	0.0463 (3)	
H8A	0.3467	0.5047	0.7102	0.056*	
H8B	0.2175	0.5182	0.5955	0.056*	
C9	0.2778 (2)	0.38392 (11)	0.62405 (14)	0.0531 (4)	
H9A	0.1862	0.3602	0.5770	0.064*	
H9B	0.3364	0.3884	0.5733	0.064*	
C10	0.34519 (14)	0.31529 (10)	0.71947 (12)	0.0404 (3)	
C11	0.33172 (14)	0.22281 (10)	0.68971 (12)	0.0413 (3)	
H11	0.2803	0.2059	0.6134	0.050*	
C12	0.39284 (14)	0.15593 (10)	0.77076 (12)	0.0399 (3)	
C13	0.47154 (13)	0.18065 (10)	0.88626 (12)	0.0391 (3)	
C14	0.48459 (14)	0.27165 (10)	0.91596 (12)	0.0429 (3)	
H14	0.5360	0.2887	0.9922	0.051*	
C15	0.42196 (14)	0.33848 (10)	0.83345 (13)	0.0437 (3)	
H15	0.4319	0.3996	0.8554	0.052*	
C16	0.6248 (2)	0.13091 (15)	1.07208 (15)	0.0661 (5)	
H16A	0.7043	0.1641	1.0636	0.099*	
H16B	0.6576	0.0755	1.1147	0.099*	
H16C	0.5771	0.1676	1.1142	0.099*	
C17	0.3105 (2)	0.03597 (13)	0.63220 (16)	0.0655 (5)	
H17A	0.2136	0.0561	0.6104	0.098*	
H17B	0.3130	−0.0294	0.6279	0.098*	
H17C	0.3553	0.0619	0.5796	0.098*	
N	0.15832 (12)	0.48049 (8)	0.73455 (10)	0.0398 (3)	
O1	0.08318 (15)	0.54928 (9)	0.89196 (10)	0.0632 (3)	
O2	0.32561 (12)	0.57504 (8)	0.88559 (10)	0.0597 (3)	
O3	0.38351 (13)	0.06447 (7)	0.74855 (10)	0.0576 (3)	
O4	0.52907 (12)	0.10956 (8)	0.95905 (9)	0.0554 (3)	
S	0.17728 (4)	0.56558 (3)	0.82594 (3)	0.04282 (12)	

### Atomic displacement parameters (Å^2^)

**Table:**

	*U*^11^	*U*^22^	*U*^33^	*U*^12^	*U*^13^	*U*^23^
C1	0.0437 (7)	0.0361 (7)	0.0379 (7)	0.0028 (5)	0.0145 (5)	−0.0021 (5)
C2	0.0480 (8)	0.0537 (9)	0.0563 (9)	0.0072 (7)	0.0267 (7)	0.0050 (7)
C3	0.0517 (9)	0.0619 (10)	0.0654 (10)	0.0210 (8)	0.0212 (8)	0.0074 (9)
C4	0.0664 (10)	0.0433 (9)	0.0581 (9)	0.0135 (7)	0.0207 (8)	0.0087 (7)
C5	0.0618 (10)	0.0416 (8)	0.0678 (11)	−0.0012 (7)	0.0302 (8)	0.0063 (8)
C6	0.0408 (7)	0.0415 (8)	0.0624 (9)	0.0015 (6)	0.0188 (6)	0.0014 (7)
C7	0.0508 (8)	0.0637 (11)	0.0547 (9)	−0.0121 (8)	0.0066 (7)	−0.0042 (8)
C8	0.0567 (8)	0.0405 (8)	0.0447 (8)	0.0030 (6)	0.0202 (7)	0.0036 (6)
C9	0.0715 (10)	0.0485 (9)	0.0413 (8)	0.0107 (8)	0.0209 (7)	−0.0004 (7)
C10	0.0421 (7)	0.0416 (8)	0.0407 (7)	0.0026 (6)	0.0175 (5)	−0.0027 (6)
C11	0.0421 (7)	0.0450 (8)	0.0355 (7)	0.0008 (6)	0.0107 (5)	−0.0069 (6)
C12	0.0386 (6)	0.0399 (7)	0.0404 (7)	0.0005 (5)	0.0115 (5)	−0.0052 (6)
C13	0.0341 (6)	0.0463 (8)	0.0366 (7)	0.0022 (5)	0.0108 (5)	−0.0026 (6)
C14	0.0356 (6)	0.0515 (8)	0.0391 (7)	−0.0031 (6)	0.0083 (5)	−0.0107 (6)
C15	0.0434 (7)	0.0401 (8)	0.0481 (8)	−0.0037 (6)	0.0153 (6)	−0.0093 (6)
C16	0.0598 (10)	0.0829 (13)	0.0438 (8)	0.0087 (9)	0.0000 (7)	0.0054 (9)
C17	0.0789 (12)	0.0467 (9)	0.0563 (10)	0.0028 (8)	0.0009 (9)	−0.0181 (8)
N	0.0436 (6)	0.0363 (6)	0.0381 (6)	−0.0006 (5)	0.0110 (5)	−0.0007 (5)
O1	0.0888 (9)	0.0641 (8)	0.0482 (6)	0.0084 (6)	0.0383 (6)	0.0079 (6)
O2	0.0581 (7)	0.0543 (7)	0.0491 (6)	0.0022 (5)	−0.0080 (5)	−0.0059 (5)
O3	0.0729 (8)	0.0390 (6)	0.0486 (6)	0.0019 (5)	0.0021 (5)	−0.0087 (5)
O4	0.0631 (7)	0.0524 (7)	0.0422 (6)	0.0095 (5)	0.0048 (5)	0.0015 (5)
S	0.0521 (2)	0.0410 (2)	0.03312 (18)	0.00382 (15)	0.01030 (14)	0.00021 (14)

### Geometric parameters (Å, º)

**Table:**

C1—C6	1.383 (2)	C10—C15	1.381 (2)
C1—C2	1.383 (2)	C10—C11	1.397 (2)
C1—S	1.7604 (14)	C11—C12	1.379 (2)
C2—C3	1.382 (2)	C11—H11	0.9300
C2—H2	0.9300	C12—O3	1.3635 (18)
C3—C4	1.369 (3)	C12—C13	1.4062 (18)
C3—H3	0.9300	C13—O4	1.3631 (17)
C4—C5	1.378 (2)	C13—C14	1.375 (2)
C4—H4	0.9300	C14—C15	1.391 (2)
C5—C6	1.377 (2)	C14—H14	0.9300
C5—H5	0.9300	C15—H15	0.9300
C6—H6	0.9300	C16—O4	1.4229 (19)
C7—N	1.4638 (19)	C16—H16A	0.9600
C7—H7A	0.9600	C16—H16B	0.9600
C7—H7B	0.9600	C16—H16C	0.9600
C7—H7C	0.9600	C17—O3	1.4173 (19)
C8—N	1.4773 (19)	C17—H17A	0.9600
C8—C9	1.519 (2)	C17—H17B	0.9600
C8—H8A	0.9700	C17—H17C	0.9600
C8—H8B	0.9700	N—S	1.6317 (12)
C9—C10	1.511 (2)	O1—S	1.4254 (13)
C9—H9A	0.9700	O2—S	1.4262 (11)
C9—H9B	0.9700		
			
C6—C1—C2	120.38 (14)	C12—C11—C10	121.59 (13)
C6—C1—S	119.60 (11)	C12—C11—H11	119.2
C2—C1—S	119.92 (12)	C10—C11—H11	119.2
C3—C2—C1	119.37 (15)	O3—C12—C11	124.96 (12)
C3—C2—H2	120.3	O3—C12—C13	115.34 (13)
C1—C2—H2	120.3	C11—C12—C13	119.70 (13)
C4—C3—C2	120.51 (15)	O4—C13—C14	126.05 (12)
C4—C3—H3	119.7	O4—C13—C12	115.10 (12)
C2—C3—H3	119.7	C14—C13—C12	118.85 (13)
C3—C4—C5	119.85 (15)	C13—C14—C15	120.89 (13)
C3—C4—H4	120.1	C13—C14—H14	119.6
C5—C4—H4	120.1	C15—C14—H14	119.6
C6—C5—C4	120.59 (16)	C10—C15—C14	120.93 (14)
C6—C5—H5	119.7	C10—C15—H15	119.5
C4—C5—H5	119.7	C14—C15—H15	119.5
C5—C6—C1	119.30 (14)	O4—C16—H16A	109.5
C5—C6—H6	120.4	O4—C16—H16B	109.5
C1—C6—H6	120.4	H16A—C16—H16B	109.5
N—C7—H7A	109.5	O4—C16—H16C	109.5
N—C7—H7B	109.5	H16A—C16—H16C	109.5
H7A—C7—H7B	109.5	H16B—C16—H16C	109.5
N—C7—H7C	109.5	O3—C17—H17A	109.5
H7A—C7—H7C	109.5	O3—C17—H17B	109.5
H7B—C7—H7C	109.5	H17A—C17—H17B	109.5
N—C8—C9	112.04 (13)	O3—C17—H17C	109.5
N—C8—H8A	109.2	H17A—C17—H17C	109.5
C9—C8—H8A	109.2	H17B—C17—H17C	109.5
N—C8—H8B	109.2	C7—N—C8	114.72 (12)
C9—C8—H8B	109.2	C7—N—S	114.70 (11)
H8A—C8—H8B	107.9	C8—N—S	115.88 (9)
C10—C9—C8	116.64 (13)	C12—O3—C17	117.65 (13)
C10—C9—H9A	108.1	C13—O4—C16	117.44 (13)
C8—C9—H9A	108.1	O2—S—O1	119.54 (8)
C10—C9—H9B	108.1	O2—S—N	106.98 (7)
C8—C9—H9B	108.1	O1—S—N	106.87 (7)
H9A—C9—H9B	107.3	O2—S—C1	108.34 (7)
C15—C10—C11	118.04 (13)	O1—S—C1	108.16 (7)
C15—C10—C9	124.05 (14)	N—S—C1	106.21 (6)
C11—C10—C9	117.90 (13)		
			
C6—C1—C2—C3	−0.6 (2)	C11—C10—C15—C14	0.3 (2)
S—C1—C2—C3	−177.00 (13)	C9—C10—C15—C14	−178.10 (14)
C1—C2—C3—C4	1.0 (3)	C13—C14—C15—C10	−0.1 (2)
C2—C3—C4—C5	−0.5 (3)	C9—C8—N—C7	−67.78 (17)
C3—C4—C5—C6	−0.4 (3)	C9—C8—N—S	154.94 (11)
C4—C5—C6—C1	0.8 (3)	C11—C12—O3—C17	2.8 (2)
C2—C1—C6—C5	−0.2 (2)	C13—C12—O3—C17	−177.37 (15)
S—C1—C6—C5	176.16 (12)	C14—C13—O4—C16	−7.8 (2)
N—C8—C9—C10	−62.34 (19)	C12—C13—O4—C16	171.97 (14)
C8—C9—C10—C15	−18.9 (2)	C7—N—S—O2	176.93 (11)
C8—C9—C10—C11	162.67 (14)	C8—N—S—O2	−45.78 (12)
C15—C10—C11—C12	−0.1 (2)	C7—N—S—O1	47.78 (13)
C9—C10—C11—C12	178.44 (14)	C8—N—S—O1	−174.93 (10)
C10—C11—C12—O3	179.51 (14)	C7—N—S—C1	−67.51 (12)
C10—C11—C12—C13	−0.4 (2)	C8—N—S—C1	69.77 (11)
O3—C12—C13—O4	0.95 (19)	C6—C1—S—O2	32.79 (14)
C11—C12—C13—O4	−179.17 (13)	C2—C1—S—O2	−150.81 (13)
O3—C12—C13—C14	−179.30 (13)	C6—C1—S—O1	163.73 (12)
C11—C12—C13—C14	0.6 (2)	C2—C1—S—O1	−19.87 (15)
O4—C13—C14—C15	179.37 (14)	C6—C1—S—N	−81.85 (13)
C12—C13—C14—C15	−0.3 (2)	C2—C1—S—N	94.55 (13)

### Hydrogen-bond geometry (Å, º)

Cg2 is the centroid of the phenyl plane C10–C15.

**Table:**

*D*—H···*A*	*D*—H	H···*A*	*D*···*A*	*D*—H···*A*
C14—H14···O2^i^	0.93	2.60	3.380 (2)	142
C8—H8*A*···O3^ii^	0.97	2.71	3.620 (2)	156
C4—H4···O1^iii^	0.93	2.65	3.369 (2)	135
C3—H3···*Cg*2^iv^	0.93	2.91	3.661 (2)	139
C6—H6···*Cg*2^ii^	0.93	3.05	3.827 (8)	123

Symmetry codes: (i) −*x*+1, −*y*+1, −*z*+2; (ii) −*x*+1, *y*+1/2, −*z*+3/2; (iii) *x*, −*y*+3/2, *z*−1/2; (iv) −*x*, *y*−1/2, −*z*+1/2.
